# Recording and communicating uncertainty in science: how geologists manage variability in spatial data

**DOI:** 10.1186/s41235-025-00682-x

**Published:** 2025-10-24

**Authors:** Cristina G. Wilson, Madelyn Sadler, Jacob Lader, Courtney Sheckler, Thomas F. Shipley

**Affiliations:** 1 Collaborative Robotics and Intelligent Systems Institute, Oregon State University, Corvallis, OR USA; 2 Department of Psychology, Temple University, Philadelphia, PA USA

**Keywords:** Uncertainty, Variability, Metascience, Geologic decision-making, Ensemble perception

## Abstract

All scientists must cope with variability in data to make inferences about the world. However, in observation-based geology, how scientists cope with variability is particularly consequential because it determines what become data in the first place, with observations that are deemed “too variable” potentially being ignored or minimized. Here, across three experiments with 97 geologists, we assess (i) how variability impacts their willingness to turn an observation into data by recording it and their willingness to share data by publishing it, and (ii) whether scientists can make inferences from variable observations and how the accuracy of their inferences is impacted by level of variability. Geologists were presented with arrays of disciplinary data representing the orientation of planar features within a rock formation, where orientation variability was systematically manipulated. Results showed substantial individual differences in criterion tolerance of variability: high-criterion individuals perceived low-to-moderate degrees of variability as more noise than signal and were never willing to publish high variability data (and often not willing to record it), while those with low criterion perceived low-to-moderate degrees of variability as more signal than noise and were always willing to record high variability data (and often publish it). Regardless of tolerance for variability, geologists overall were good at making accurate orientation estimates from variable data, even at the highest levels of variability employed in the study. Together, these results imply there may be situations where scientists avoid recording or publishing variable data, despite being able to draw meaningful conclusions from such data.

## Introduction

The job of a scientist is to use data and observations to make inferences about the world. One persistent challenge to doing so is the presence of uncertainty. Uncertainty that is *internal* and arises from a lack of knowledge can be reduced or eliminated, but uncertainty that is *external* and arises from inherent variability cannot be (Kahneman & Tversky, 1982; Li et al., 2012; Schunn & Trafton, 2013). While all scientists must cope with variability, and the problem of extracting signal from noise, different sciences have very different levels of variability and types of signals to identify. In this paper, we focus on variability in field geology and its impact on science practice.

In field geology, signals are spatiotemporal and are obscured by variability from multiple geologic processes that combine to transform the Earth’s surface over large time spans. Geologists generally extract signals from noise through observation. Some observations are recorded, turning them into data, while others are retained only in the mind of the geologist: these may inform or bias future data, but from the perspective of the science community they are effectively lost. Thus, unlike other experiment-based sciences, where variability must be managed once data are collected, *in observation-based geology, how variability is managed determines what become data in the first place*. Furthermore, techniques for managing variability in observation are applied at the individual level and likely depend on one’s experience, mentor/community standards (Wilson et al., 2021), sensitivity to variability, and other factors: there are no overt disciplinary rules that exist for coping with variability in observations like there are for coping with variability in data analysis and communication.

Readers from the experimental sciences where standards of evidence require considerations of variability in planning (e.g., power analyses) and confidence in conclusions (e.g., effect sizes) may find concerning the practice in geology of selecting data to record and relying on small numbers of observations for conclusions. The contrasting practices may reflect differences between Earth science and experimental or social science data in the vulnerability to noise in small samples (Shipley et al., 2025). Regardless of the cause, in practical terms geology has a long history where these practices have been successful in developing an understanding of Earth processes, as measured by the stability of data (e.g., geologic maps made 50–100 years ago are still cited and relied on for field work), and in successfully applying the theories to make economically valuable predictions (e.g., locating ore deposits and hydrocarbon reservoirs). Therefore, it is not the case that geologists’ management of variability is leading to poor science outcomes, but rather that the nature of the science makes *how* one manages variability incredibly consequential for data collection decision-making.

The goal of the study presented here was to determine how geologists’ perceive different degrees of variability in disciplinary observations and how it impacts their science judgments. This was accomplished across three experiments with 97 total geologists. In Experiment 1, we assess how levels of variability affect geologists’ willingness to turn an observation about the world into data by recording it and their willingness to share that data by publishing it. In Experiments 2 and 3, we again characterize choices to record and publish data for a range of variability and also assess whether geologists can make inferences from variable observations and whether the accuracy of their inferences is low at levels of variability they would not record or publish. Experiments 2 and 3 differ in the order of study material presentation, to ensure results were not driven by making inferences about variable data prior to judgments of willingness to record and publish. In the next section, we review the cognitive science literature on perceiving variability and coping with uncertainty, but first we provide some context for the non-geologist reader on how variability is communicated in disciplinary data products, i.e., geologic maps.

## Background

### Visualizing variability in geologic maps

Mapping is one of the ways that geologists collect and communicate data about observations. Geologic maps present data at different scales about rock formation types and contacts (changes in rock type), as well as structures like faults and folds. To characterize the structures, geologists collect a variety of 3D data. In the current study, we utilize a common type of 3D data, *strike-dip measurements*, to assess geologists’ perceptions of variability.

Strike-dip measurements capture the orientation of planar features within a formation (e.g., bedding, which is the boundary between sediment layers that became rock). Many planar features were originally horizontal but have changed orientation as the rocks have been deformed by tectonic processes. Strike is the intersection of the plane of the feature with a horizontal plane, and dip is how much the plane diverges from horizontal. These are presented as T-shaped symbols on a geologic map, where the top of the T, the longer line, indicates the strike direction and the shorter, perpendicular line indicates the dip direction with a number presenting the dip angle.

**Fig. 1:**
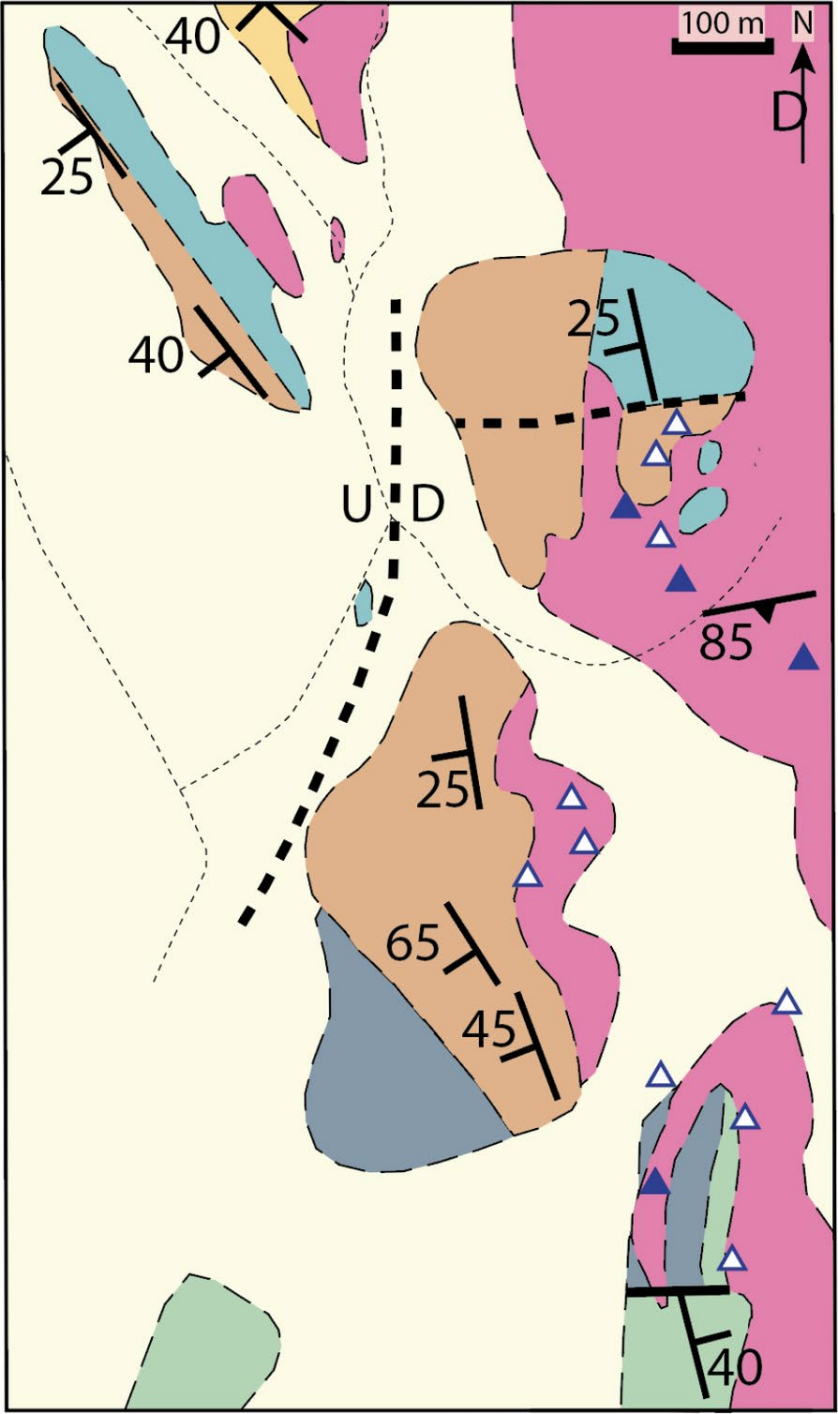
A portion of a geological map in the White Inyo mountains. Different colors are interpretations of the data indicating the mapper’s models of spatial distributions of different assemblages of rocks. The light yellow represents younger rocks and unconsolidated material that cover the subsurface rocks which were the focus of mapping and generally have lower uncertainty than the yellow region. The thick dotted lines represent where the mapper infers there are likely to be faults. The triangles are locations where rock assemblages were observed but orientation could not be measured. The T-shaped strike-dip symbols communicate the orientation of a measured plane and therefore indicate where rock type and orientation data were recorded. In this map, only a single orientation is reported at a location. Likely there were many possible planes that could have been measured at the location. This sole observation likely represents the mapper’s assessment of a representative plane. Some mappers will record more than one observation at a location in which case that data might be presented in a stereonet (a visualization where 3D planar orientation is represented as a single point) to convey variability. Figure adapted from Nelson et al., 2023

As illustrated by the geologic map in Fig. 1, maps have variability within their strike-dip measurements. This variability can be because of systematic patterns reflecting geologic processes (e.g., the rocks in the region have been folded so the dip direction reverses at the center of the fold), local variability in a feature of the rock (e.g., bedding), movement of the rock if it became detached from the subsurface, or measurement error. To arrive at an interpretation of the rock structures in an area, and from that a model of the history of how rocks have moved in an area, geologists focus their analyses on the systematic patterns (signal) and ignore (e.g., look through) the noise of the local variations. We leverage geologists’ familiarity with the practice of visually extracting patterns from variable data in the current study by having them make judgments about arrays of strike-dip symbols with different degrees of variability in strike. In particular, we focus on strike because strike is isomorphic to the spatial information in the world, whereas dip partially relies on a number to convey spatial information). The ability to pick out both an overall trend and how the items as a collective vary is known in the cognitive science literature as ensemble perception.

### Ensemble perception

Prior studies on ensemble perception provide participants an array of items and ask them to judge feature probabilities, such as the mean or the variance. Items can be presented simultaneously (Morgan et al., 2008) or over time (Witt, 2019), and range in type: e.g., oriented line segments (Norman et al., 2015), facial displays of emotion (Haberman et al., 2015), hurricane trajectories (Padilla et al., 2017; Liu et al., 2018; Padilla et al., 2020). Results across studies show people are generally good at estimating means from an ensemble. Particularly relevant to our study, past work has shown people are able to estimate average line orientation from multi-item arrays presented simultaneously (Parkes et al., 2001).

People also tend to be good at assessing variability from ensembles (Morgan et al., 2008; Lau & Brady, 2018; Solomon et al., 2010; Solomon et al., 2011). For example, a body of research on visualizations of hurricane paths has shown, among non-experts, ensembles lead to better understanding of the uncertainty in model predictions (Padilla et al., 2017, 2020) than summary statistics of variability (Ruginski et al., 2016). However, people can also overweight items in ensembles (e.g., a hurricane path impacting a point of interest), particularly when the total number of items in the ensemble is smaller (e.g., 1 out of 9 paths impact point of interest versus 1 out of 33). Liu et al. (2018) found more than 15 hurricane paths were needed for people to have well-calibrated uncertainty. In the current study, because we use arrays of 300 + strike-dip symbols for ensemble perception, any visual–spatial bias (Padilla et al., 2021) toward single items should be minimal. More pertinent to our work is research showing different sensitivities to variability can lead people to overestimate or underestimate the exact degree of variability within an array. Witt (2019) found non-geologist participants overestimated the variability in arrays of oriented lines (similar to strike-dip symbols) presented one at a time in rapid sequence. Participants estimated the line trend and range of orientations by selecting the best match from among a set of static images of line sets. Participants overestimated the variability in the animated sets of lines, matching them to static images of line sets that were more variable than what they had experienced, and this effect was robust with respect to the orientation of the lines. There was also some evidence that overestimation increased with greater ranges of line orientation; however, in the study, lines only differed in orientation by a maximum of 8°, which is limited relative to geologic data where pure noise will range +—180°.

In contrast, ensemble perception studies of trajectory prediction, where participants estimate the final position of moving points, have found that people consistently underestimate variability. For example, Pugh et al. (2018) asked participants how many endpoints would have fallen within a given radius and found they underestimated the variability by up to 30%, reporting more endpoints within the given area than they had actually seen in the initial animations. Other studies of trajectory prediction have found similar tendencies to underestimate variability, with the degree of underestimation increasing as variability increases (Wickens et al., 2020).

Given that trajectory prediction places a high demand on working memory, underestimation of variability could reflect failure to encode or retain endpoints that greatly differ from the mean (Kareev et al., 2002; Padilla et al., 2021). However, ensemble perception of temporally presented lines (Witt, 2019) would also be demanding on working memory and resulted in overestimation of variability. In the current study, we use static arrays of strike-dip symbols for ensemble perception, so any underestimation or overestimation of variability that occurs cannot be driven by constraints on working memory. Instead, we expect sensitivity to variability to be the result of individual or disciplinary strategies for coping with scientific uncertainty.

### How scientists cope with uncertainty & variability

Scientists experience external sources of uncertainty in data directly—as they collect or aggregate data—and indirectly—as it is communicated to them in papers or talks by other scientists. While data from other researchers would seem like a rich source of information about variability, it may be that uncertainty is not broadly communicated, particularly in the context of translational reports about data. A survey of 90 visualization experts (who construct data visualizations for scientists, the general public, and policy makers), reported they avoid uncertainty when communicating data due to field standards or challenges explaining the uncertain data to others (Hullman, 2019). This could lead scientists to underestimate the degree of variability present in others’ data, which, in turn, could lead them to ignore or minimize variability in their own data. In line with the latter, Boukhelifa et al. (2017) found data workers most often report adopting the explicit strategy of *minimizing* variability (e.g., filtering outliers and aggregating data). Other common strategies reported include *ignoring* variability (e.g., excluding data when sources conflicted and ignoring missing data) or *understanding* variability (e.g., statistical quantification of uncertainty and including meta-data about the reliability of the sources).

Applying the findings of Boukhelifa et al. (2017) to geology, scientists commonly *minimize* uncertainty by publishing an observation at one point based on a visual estimate that it is representative of the surrounding rocks. Alternatively, geologists sometimes try to *understand* the variability by collecting multiple observations at a single location (Wilson et al., 2021), and using directional (e.g., circular, spherical) statistics (Downs, 1972) to aggregate across observations (Borradaile & Borradaile, 2003) and test hypotheses (Davis & Titus, 2017). Geologists *ignore* variability in one of three ways: they choose not to make a measurement, they choose not to record a particular observation, or they choose not to share it with the science community by publishing it. Geologists chose not to make a measurement in cases where the variability is too high (e.g., cross-bedding in a sandstone), there is an indication that the original geometry has been modified (e.g., slumping), or the rock type does not preserve good features (e.g., bedding in massive limestones). If a geologist makes a measurement but chooses not to record it, the observation is retained only in their mind; if they choose not to publish it, the observation is kept private in a field journal or digital database. In either case, the observation is dark data, effectively lost to the science community.

Here, across three experiments, we assess geologists' propensity to ignore variability in strike-dip data arrays by choosing not to record or publish it. In Experiments 2 and 3, we again characterize choices to record and publish for a range of strike-dip variability and also assess geologists’ ability to accurately estimate the representative strike orientation of these arrays. Such an ensemble estimation skill would be one strategy that could minimize variability in recorded data. In Experiment 2, participants make orientation estimates prior to record and publish judgments, whereas in Experiment 3, participants make record and publish judgments first.

## Experiment 1

The goal of Experiment 1 was to assess how geologists’ perceptions of different levels of variability impact their willingness to turn an observation about the world into data. To this end, we presented geologists with a range of variability of strike in arrays of strike-dip measurements and asked them to report their perceived variability, and their willingness to record the data and share that data by publishing it.

### Methods

#### Design and variables

This experiment used a within-subjects design in which participants were asked to make judgements of 10 arrays of strike-dip symbols with different strike array variability. The independent variable was strike array variability. To isolate uncertainty to the variability in strike, participants were informed that the rock in all cases was steeply dipping (for shallow dips, strike value is sensitive to small differences in measurement relative to horizontal). Thus, these arrays represented the repeatability of an observation made at a location. Strike array variability was sampled from a normal distribution, and images were generated using MATLAB as described in detail below. The strike array variability ranged from 5° to 42.8°. This range was determined through discussions with geologist collaborators about the low and high end of variability to be expected when an expert measures strike at the same location multiple times, and through pilot testing with sample arrays; 5° was our collaborators’ estimate of variability in recording strike in the field under ideal conditions.

The dependent variables were judgment of willingness to record the observation, judgment of willingness to publish the observation, subjective rating of variability, and categorical judgment of variability. Possible responses for the judgments of willingness to record and publish were Yes, Unsure, and No. Subjective rating of variability was on a scale from 0 (no variability) to 100 (random noise). Lastly, the categories for descriptors of variability were Unreliable, Permissive, Suggestive, Presumptive, Compelling, and Certain. Each category term was accompanied by a brief description of the meaning based on terms employed by Bateman et al. (2022) to help students communicate about uncertainty in data. The terms themselves come from geology practice and the accompanying descriptions were provided to participants in case they were unfamiliar with usage.

#### Hypotheses

We hypothesized that there would be a nonlinear, sigmoidal relationship between willingness to record or publish data across different levels of strike array variability (**H1**), i.e., that participants would be willing to record or publish data when the strike array variability was low, but upon reaching a threshold of greater variability, would no longer be willing to record or publish. We also hypothesized that the threshold of strike array variability would be greater for willingness to record than for willingness to publish (**H2**), i.e., that participants would be willing to record data at higher levels of strike array variability than they would be willing to publish. In addition to testing these hypotheses, we explored the subjective rating of variability that participants associated with the threshold at which they were no longer willing to record or publish. This allowed us to characterize how participants perceived data they were no longer willing to record or publish, on a continuum of ideal signal to complete noise. Finally, we hypothesized that there would be good agreement among participants in the strike array variability associated with each categorical descriptor of variability (**H3**). Our proposed categories are based on work showing that people can consistently report their level of experience on a psychological dimension (Preston & Coleman, 2000). Thus, H3 follows from the claim that people have access to their uncertainty as an intensive psychological variable, and therefore, a community with experience in data variability should tend to agree in their assignment of ordinal categories of orientation variability.

#### Materials

**Fig. 2:**
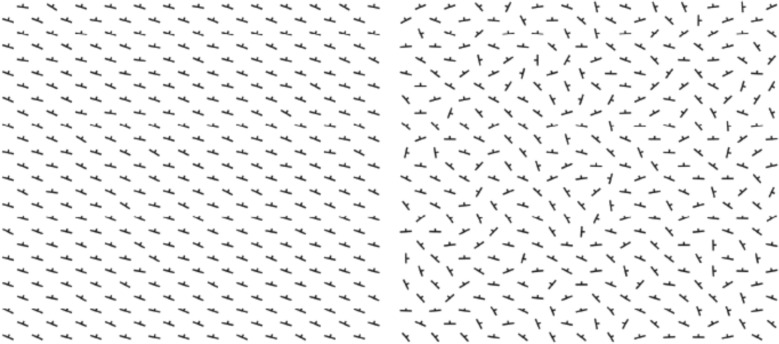
Arrays of strike-dip symbols showing 5° of variability (left) and 42.8° of variability (right)

We created 10 arrays of strike-dip symbols meant to communicate potential variation in observations of a spatial geological feature in the field (see Fig. 2). Stimuli were generated using MATLAB Version: 9.12.0 (R2022a) Update 4 (The MathWorks Inc., 2022). The 10 arrays had different strike variability each sampled from a normal distribution. The lowest strike array variability had a standard deviation of 5°. From there, strike array variability increased by 4.2° steps up to a maximum standard deviation of 42.8°. The specific values of strike array variability used were: 5°, 9.2°, 13.4°, 17.6°, 21.8°, 26°, 30.2°, 34.4°, 38.6°, and 42.8°.

The MATLAB script generated 338 strike symbols whose orientation was randomly sampled from a normal distribution defined by the standard deviation for each array. The sampling was constrained by rejecting values outside of + 3 or −3 standard deviations. The resultant value was added to the starting orientation of 25°. For these arrays, 25° avoided creating visually distracting linear patterns by staggering the strike symbols above and below each symbol.

All arrays were generated as a 900 pixel wide by 850 pixel tall figure. Strike lines were 26 pixels long with a dip line of 4 pixels plotted orthogonal to strike orientation. The strike-dip symbols were arranged in pairs in a 13 by 13 checkerboard pattern with one at the upper left corner and one in the center of each cell. Thus, there was a space in the horizontal and vertical direction between every generated symbol in the figure. To avoid alignments of adjacent strike symbols, the x and y coordinates of each symbol were displaced by a random number of pixels up to 25 horizontally and 25 vertically, which offset the symbols without moving any so far that they would overlap.

#### Procedure

Participation occurred through a web-based Qualtrics survey. All participants were first asked to provide informed consent and confirm that they were completing the survey on a desktop or laptop computer (and not a mobile device). Once this was completed, participants received a short introduction explaining what the stimuli represented and what kinds of questions they would be asked. Then participants were presented with the 10 arrays of strike-dip symbols one at a time. Alongside the 10 arrays was a short description reminding the participant of what the array does and does not represent. Each array was also captioned as being a location (e.g., Location A) to reinforce the idea that it simulated a single location with multiple observations. All arrays were shown in the same order for all participants from least to greatest strike array variability.

Each array and its description were shown on the same page as the questions, and participants were not able to go back to previous arrays/questions once they had moved on to the array image. The first question asked participants whether they would record an observation with the level of strike variability they saw in the given image. If participants responded Yes, or Unsure to this question, they were asked whether they would include the observation in data they published. For scoring purposes, anyone who did not respond Yes to the record question was scored as a No for the publish question, because an unrecorded observation cannot be published. Regardless of whether they would record the observation, participants were then asked to select among a set of six categorical descriptors of variability for the displayed array: Unreliable (*this datum provides no evidence for an interpretation that relies on the rocks in the region having the orientation of this observation*), Permissive (*it is possible this datum could provide evidence in favor of an interpretation…*), Suggestive (*this datum provides some evidence for an interpretation…*), Presumptive (*this datum provides evidence that is more likely than not in favor of an interpretation…*), Compelling (*this datum provides strong evidence in favor of an interpretation…*), Certain (*this datum provides a direct and resolvable link to an interpretation…*). The final question asked participants to rate the variability using a scale from 0 (no variability) to 100 (random noise). After all arrays and associated questions were presented, demographic information was collected, including area of specialization, years of experience, level of education or highest appointment, and primary professional environment (i.e., industry or academia).

#### Participants

Forty-five experts in a variety of geology fields participated in this experiment and had their data analyzed. All participants had at least a Bachelor’s degree and at least 5 years of experience post-bachelor’s (*M* = 23.07, *SD* = 11.38). The majority of participants (*n* = 29) reported specializing in structural geology and tectonics, with a few (*n* = 3) specializing in mineralogy, geochemistry, petrology, and volcanology, and no more than 2 specializing in any other subarea. Most participants also reported primarily working in academia (*n* = 43) while 1 reported primarily working in industry and 1 reported working in both. Participants were recruited to the study through email lists, organizational listservs, and recruiting emails sent to geologists with existing relationships with the study personnel with requests to forward to colleagues. Data were collected from April 2023 to July 2023. The study protocol, recruitment, and consent procedures were approved by the Temple University Institutional Review Board.

#### Data analysis

All statistical analyses for this study were completed in RStudio version 4.2.0 (R Core Team, 2022). Prior to running any analyses, we cleaned the data by removing participants based on the following criteria: they were not 18 years of age or older (2 participants excluded), they did not complete the questions on a laptop or computer (none excluded), they did not complete at least 75% of the survey questions (none excluded), they had a mean negative slope for their subjective rating of variability across strike array variability levels (2 participants excluded). For the latter, since strike array variability levels increase in variability, a mean negative slope for a participant’s subjective rating of variability suggests misuse of the rating tool or a misunderstanding of the task. We removed 4 participants through this cleaning procedure from the 49 total who participated, and ran all subsequent analyses on the 45 remaining participants.

In order to evaluate the relationships between strike array variability and the willingness to record and publish an observation, we fit a log-logistic sigmoid curve to the group’s data to willingness to publish and willingness to record data. Because we know the possible range of responses, we can define the bounds for the curve at the lower asymptote, *a* = 1, and the upper asymptote, *d* = 3. To test **H1**, we use a lack of fit test to evaluate whether there is a natural nonlinear sigmoidal relationship between participants’ willingness to record or publish data and the different levels of strike array variability.

To test **H2**, we refer to the inflection point and slope of the log-logistic sigmoid curve. The inflection point of this curve, *c*, represents the level of strike array variability at which participants were 50% likely to publish or record observations. In other words, it represents the point where participants were Unsure, shifting from predominantly Yes responses to predominantly No responses. The normalized slope around the inflection point, *b*, indicates how quickly their willingness declined, which is representative of the degree of agreement in judgments: larger slopes mean greater numbers of participants shift from Yes to No at the inflection point, whereas smaller slopes mean there is a more gradual shift. We also calculated the mean and standard deviation of participants' subjective rating of variability at the inflection point to characterize how participants perceived data they were no longer willing to record or publish, on a continuum of ideal signal to complete noise.

To test **H3**, we determined the range of strike array variability encompassing each of the categorical descriptors of variability by calculating the median and IQR of each level of strike variability for each categorical descriptor. This shows visual trends of agreement among participants’ classifications of each descriptor of variability. To test participants’ agreement, we computed Fleiss’ kappa, which evaluates the degree of agreement between two or more raters on a categorical scale. However, Fliess’ kappa does not account for similarity between categories, which means two participants who rated a strike array variability as Certain and Compelling have the same level of disagreement as two participants with Certain and Unreliable ratings. To account for this, we also conducted a simple linear regression analysis to test whether strike array variability significantly predicted participants’ categorical descriptor selection using numeric representations of the categorical descriptors of variability (Unreliable = 1, Certain = 6). Finally, we explored agreement between categorical descriptors of arrays and willingness to publish by computing the percentage of participants who responded Yes (would publish) when labeling arrays Certain and Compelling, and responded No (would not publish) when labeling arrays Permissive or Unreliable.

### Results

**Fig. 3:**
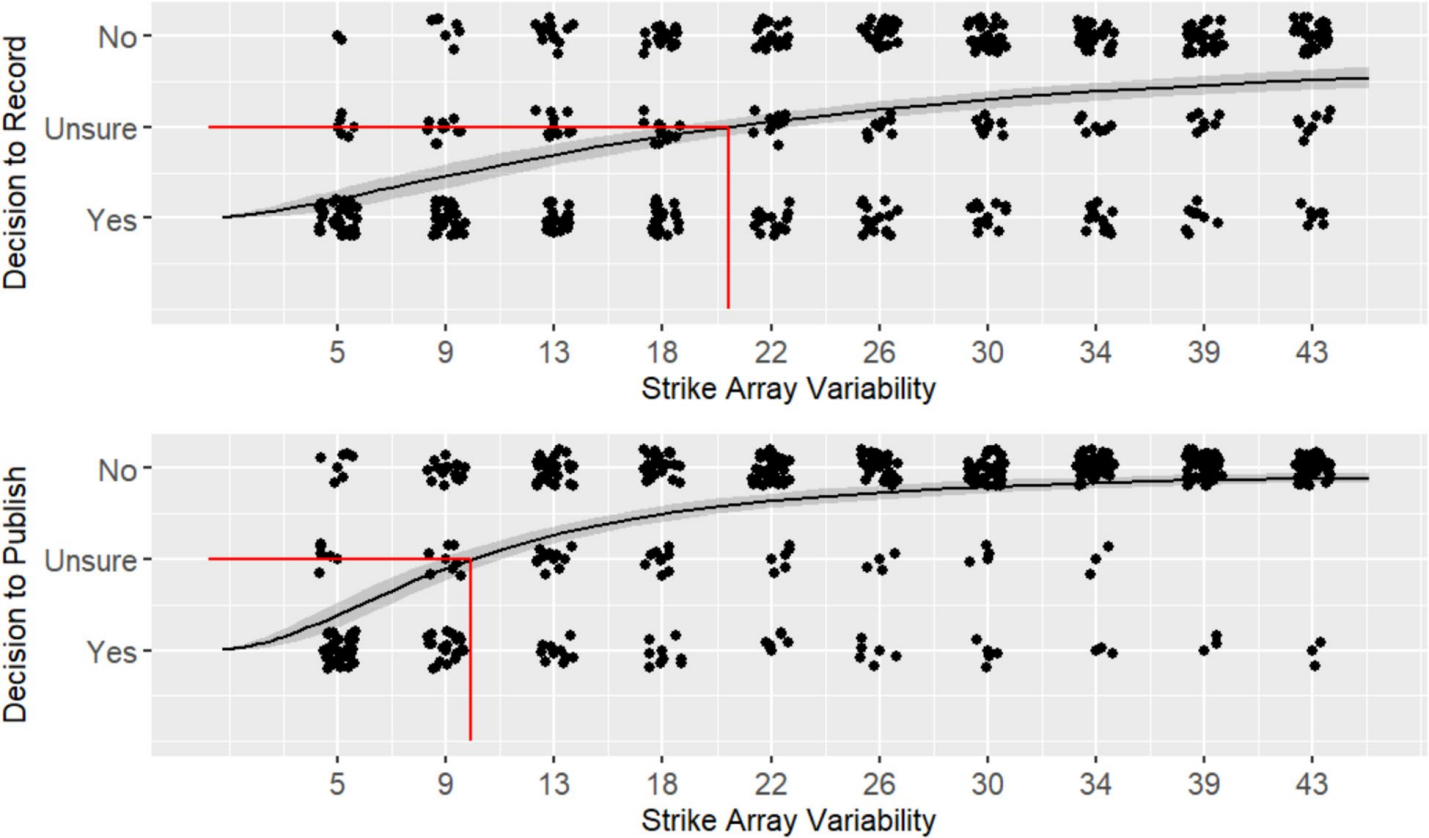
Willingness to record (top) and publish (bottom) across strike array variability level for Experiment 1. Participant response data, represented by the black dots, is jittered for ease of viewing. A sigmoid curve is fit to the response data, represented by the black line, with a 95% confidence interval, represented by the gray shadow around the line. The inflection point of the curve, represented by the red line, indicates the strike array variability level where participants were Unsure, shifting from predominantly Yes responses to predominantly No responses

For **H1,** a lack of fit test was non-significant for both willingness to record (*F* (8) = 0.128, *p* = 0.998), and willingness to publish (*F* (8) = 0.128, *p* = 0.998), indicating the hypothesis is supported and the log-logistic sigmoid curve is a good fit for the data (see Fig. 3). For willingness to record, the residual standard error (RSE) was 0.80 (448 degrees of freedom), and for willingness to record the RSE was 0.67 (444 degrees of freedom).

For **H2**, we compared the inflection point of the curves at which willingness to record and publish began to decrease. Results supported the hypothesis that the threshold for participants’ willingness to record (*c* = 20.14) would be greater (*t* (64) = 7.36, *p* < 0.001, *d* = 1.55) then their willingness to publish (*c* = 10.15), indicating participants were more tolerant of variability when recording, as opposed to publishing data. Notably, these inflection point values reflect relatively low degrees of strike array variability compared to the full range of variability presented; put another way, participants in Experiment 1 reported being unwilling to record 6 of the 10 arrays presented and unwilling to publish 8 of the 10. The slope at the inflection point for willingness to publish (*b* = −1.99) was greater (*t* (88) = 1.71, *p* = 0.045, *d* = 0.36) than the slope at the inflection point for willingness to record (*b* = −1.53). That willingness to publish declined more rapidly than willingness to record indicates participants were in greater agreement about the level of variability at which data became unpublishable than they were in agreement about the level of variability at which data became unrecordable.

In alignment with these findings, participants’ average subjective rating of variability at the respective thresholds was greater for the decision to record (*M* = 71.02, *SD* = 19.44) than the decision to publish (*M* = 54.02, *SD* = 20.38). This further supports that participants are more tolerant of variability when recording data than when publishing. It also suggests that they perceive the relatively low strike array variability with a standard deviation of ~ 10° (inflection point for willingness to publish) and ~ 20° (inflection point for willingness to record) as being closer to random noise than to signal on the subjective scale.

**Fig. 4:**
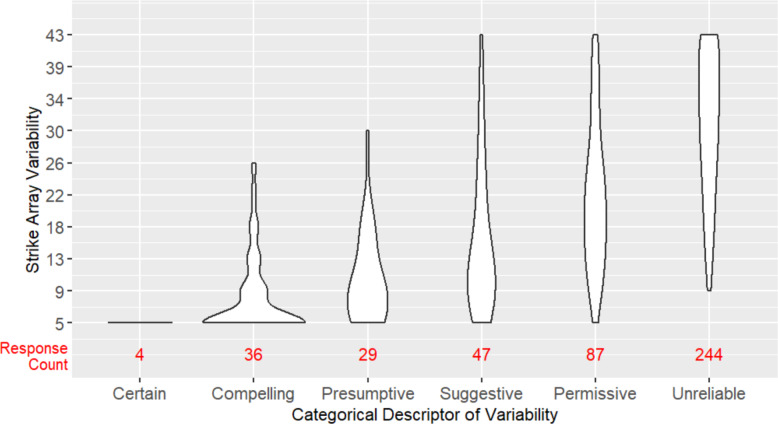
Categorical descriptors of variability used to label strike array variability levels for Experiment 1. The width of each violin plot shows the approximate relative frequency of descriptor selections at that level of strike array variability. Response counts (in red) give the absolute frequency of descriptor selection

For **H3**, we explored the agreement among participants in the strike array variability associated with each categorical descriptor of variability. As shown in Fig. 4, median strike array variability does increase progressively moving from Certain to Unreliable, but the distributions of each descriptor remain spread across levels of strike array variability, suggesting imperfect agreement. A Fleiss kappa analysis showed significant “slight agreement” (*k* = 0.178*, p* < 0.001) according to Fleiss classification (Fleiss et al., 2003). A simple linear regression analysis, however, showed increasing strike array variability significantly predicted the selection of terms moving from Certain to Unreliable (*β* = −0.07, *p* < 0.001), accounting for 43% of the variation in responses (*F* (1, 445) = 337.7, *p* < 0.001). Taken together, these results provide some support for the hypothesis that there would be good agreement in the strike array variability associated with each categorical descriptor—participants seem in good agreement about the array variability associated with lower levels of uncertainty (Certain, Compelling, Presumptive, Suggestive), but widely differ in the array variability they associate with higher uncertainty terms (Permissive, Unreliable). In aggregate, participants tend to report that they would publish Certain data and Compelling data (35/40, 87.5% Yes responses to willingness to publish question), and would not publish Permissive and Unreliable data (287/331, 86.7% No responses to willingness to publish question). However, as shown in Fig. 4, the number of responses for the different descriptors of variability is heavily skewed, with participants disproportionately selecting the high uncertainty terms (Permissive, Unreliable) to label the arrays.

### Discussion

The results from Experiment 1 suggest scientists tend to have a high-criterion tolerance of variability. Most participants were only willing to record data from the 4 arrays with the lowest degree of variability (up to ~ 20° standard deviation) and only willing to publish data from the 2 lowest variability arrays (up to ~ 10°). On average, participants’ subjective ratings of variability at these record/publish thresholds were relatively high, falling above the half-way point (50) on a scale of signal (0) to random noise (100). While participants overall had a high-criterion tolerance of variability, there were clear individual differences, particularly for willingness to record. As shown in Fig. 3, and indicated by the slope at the inflection point of the sigmoid curve, the shift from being willing to record to being unwilling is much more gradual than for decisions to publish, with a number of participants still willing to record data even at the highest levels of variability.

One possible explanation for participants having higher-criterion tolerance of variability is that they are actually not able to extract a trend in strike orientation from the more variable arrays, i.e., they are choosing not to record or publish higher variability data because they cannot interpret it. Alternatively, it may be that participants are able to make accurate estimations of variable data, but simply choose not to record or publish such data. The latter is more likely given the quality of geology data, and might reflect logistical constraints in the collection of field data, i.e., devoting limited time and resources to areas where meaningful, systematic trends can be confidently observed. We evaluate participants' ability to accurately estimate the representative strike orientation from arrays in Experiment 2. If estimation accuracy is equivalent for arrays participants choose to versus not to record or publish, it would suggest that there are situations where scientists avoid recording or publishing variable data, despite being able to draw meaningful conclusions from such data.

## Experiment 2

The goal of Experiment 2 was to assess geologists’ accuracy in visually estimating a trend from data with different levels of variability and characterize how estimates of variability relates to their willingness to turn an observation about the world into data by recording it and their willingness to share that data by publishing it.

### Methods

#### Design and variables

This experiment used the same design and variables as Experiment 1, but with an added measure of participants’ strike orientation estimate. Estimates were used to compute two additional dependent variables: the error between the true average orientation of a strike array and the participants’ estimates, and the difference score between the true average orientation of a strike array and the orientation of each individual strike.

#### Hypotheses

We had two additional hypotheses to those stated in Experiment 1 (**H1—H3**), corresponding to the new dependent variables in Experiment 2. Regarding the error between the true average orientation of a strike array and the participants’ estimates, we hypothesized that error would be greater within the strike arrays that participants choose *not* to publish than the arrays that they choose *to* publish (**H4**). This hypothesis was driven by the assumption that participants’ would choose not to publish data at levels of strike array variability where they could no longer make an accurate estimate of strike orientation. Therefore, we also explored the relationship between participants’ estimate error and strike array variability.

If participants can estimate the overall trend across multiple strikes, they should be more accurate than guessing. However, one could do much better than guessing a random direction, without estimating an ensemble, by basing a guess on a single item in the array. Therefore, we compared participants’ orientation estimates to the average estimate one would expect if one were to estimate the array by choosing an item from it at random and reporting that item’s orientation. The expected error from this strategy would be the average (across all items in the array) of the unsigned difference between the item and the average trend of the array, which we refer to as difference score.

We hypothesized that the difference score would be greater than participants’ estimate error (**H5**). This hypothesis reflected our expectation that participants would be able to extract an orientation trend from the array, even at higher levels of variability, i.e., that they are not just guessing an orientation. If participants’ error is the same as the difference score, it would indicate that they are guessing by selecting a single item from the array and reporting that orientation. However, if the hypothesis is supported, it is most likely the case that participants are performing some sort of aggregation of items from the array to estimate trends in orientation. To test orientation estimation in Experiment 2 and 3 we created a set of strike-dip arrays like ones used in Experiment 1 but varied in overall orientation.

#### Materials

For Experiment 2, we created 30 new arrays of strike-dip symbols using the same MATLAB script and 10 strike variability values as in Experiment 1. The stimuli had the same number of strike-dip symbols, however in Experiment 2 the overall trend varied over 360° across arrays. To avoid the appearance of adjacent strike symbols aligning, the randomized offsets of each symbol were increased such that each was displaced by a random number of pixels up to 25 horizontally and 60 vertically. Each strike array variability value appeared 3 times under 3 different orientations (see Table 1), rather than always using the same starting orientation as in Experiment 1. We determined starting orientation values of 45° up to 157.05°, in increments of 12.45°. We chose these initial orientations so that by adding a random value between 115° and 125° and adding a random value between 235° and 245° we would have a homogeneously circularly distributed set of orientations. We subtracted 360° from any resulting orientations that were greater than 360°, which filled in the 0 to 45° range. Strike orientations were generated using a python script in Visual Studio Code version 1.86.0.

**Table 1:** Strike array variability levels and corresponding strike orientations for the 30 arrays in Experiment 2

Strike variability	Strike orientations		
5	45	169.4	285.8
9.2	57.4	176.3	294.8
13.4	69.9	189.8	313.9
17.6	82.3	205.0	335.5
21.8	94.8	213.4	322.2
26.0	107.2	226.5	342.6
30.2	119.7	237.1	358.4
34.4	14.8	132.1	251.5
38.6	25.4	144.6	261.4
42.8	40.1	157.0	272.1

**Fig. 5:**
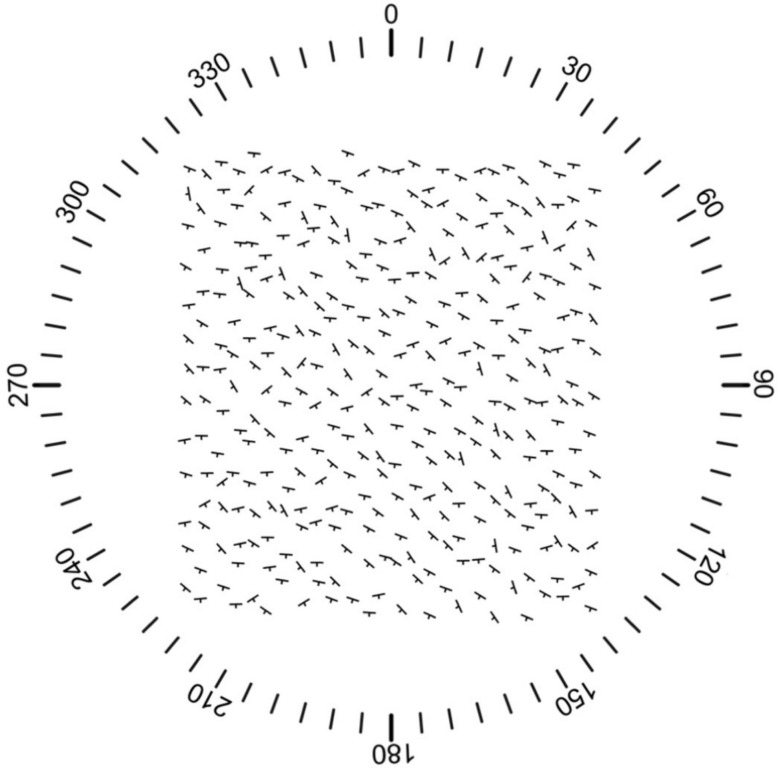
Array of strike-dip symbols showing 26° of variability with surrounding compass. The average strike trend is 107° using the right-hand rule

The MATLAB script was used to generate the new arrays of strike-dip symbols that were visually comparable to those from Experiment 1 (see Fig. 5). However, for Experiment 2, we placed a compass around each of the arrays, with clockwise poles at 0°, 90°, 180°, and 270° and with intermediary markings every 30°, to provide information about how to report the average trend of the strike lines.

#### Procedure

As in Experiment 1, participation occurred through a web-based Qualtrics survey. All participants were first asked to provide informed consent and confirm that they were completing the survey on a desktop or laptop computer (and not a mobile device). Once this was completed, participants received a short introduction explaining what the stimuli represented and what kinds of questions they would be asked. Then participants were presented with the 30 arrays of strike-dip symbols one at a time, in random order. On the same page as each array was a short description reminding the participant of what the array does and does not represent (comparable to Experiment 1), and a question asking participants to estimate the overall trend of the strike orientation in degrees (1° to 360°) using the right-hand rule, which is a community standard that uniquely defines the orientation of the line relative to dip (the strike direction is indicated by the top of the strike line relative to the dip symbol on the right side of the line).

After making the 30 strike estimates, participants were presented with a subset of 5 arrays from the 30 and asked the same questions as in Experiment 1 (willingness to record, willingness to publish, categorical judgment of variability, and subjective rating of variability). Here we employed only a subset to keep the duration of the experiment reasonable. The 5 arrays had variability values that encompassed the range used in Experiment 1: 5°, 13.4°, 21.8°, 30.2°, and 42.8°. The orientation of the array at each variability value was randomly selected from the 3 possible orientations for each participant. After responding to questions about the 5 arrays, participants completed the same demographic questions as Experiment 1.

#### Participants

Thirty experts in a variety of geoscience fields participated in this experiment and were included in the analysis. All participants had at least a bachelor’s degree and 90% had at least 5 years of experience post-bachelor’s (*M* = 23.51, *SD* = 14.74). The majority of participants (*n* = 25) reported specializing in structural geology and tectonics, with no more than 2 specializing in any other subarea. Most participants also reported primarily working in academia (*n* = 14) while 5 reported primarily working in industry, and 2 reported working in both. Participants were recruited using the same methods as Experiment 1. As participation was anonymous, we have no way of determining whether any of the participants from Experiment 1 also participated in Experiment 2. Data were collected from February 2024 to April 2024.

#### Data analysis

We used a comparable data cleaning procedure as Experiment 1, removing participants based on the following criteria: they were not 18 years of age or older (2 participants excluded), they did not complete the questions on a laptop or computer (12 participants excluded), they did not complete at least 75% of the survey questions (17 participants excluded), they had a mean negative slope for their subjective rating of variability across strike array variability levels (2 participants excluded). For Experiment 2 we also excluded participants when more than 1/2 of their estimations of overall strike array orientation had an error that differed by more than ± 2 standard deviations from the mean of actual strike array orientations—this filter picked up any participants who were reporting dip direction instead of strike as their errors were ~ 90 deg from the correct direction (2 participants excluded). We removed 35 participants through this cleaning procedure from the 65 total who participated, and ran all subsequent analyses on the 30 remaining participants.

All statistical analyses for **H1**, **H2**, and **H3** were identical to Experiment 1. To test **H4**, we ran independent sample t-tests to compare participants’ average estimate error at the strike array variability levels they would, versus would not publish. We also calculated the mean and standard deviation of participants' estimate error at all levels of strike array variability (regardless of willingness to publish), and ran a simple linear regression analysis to test if variability level significantly predicted participants’ error.

To test **H5**, we ran paired samples t-tests to compare participants’ average estimate error to the average difference score for each level of strike array variability. If error is less than the difference score, it indicates that participants are able to aggregate items from the array to estimate trend in orientation; if the error is equal to the difference score, it is consistent with participants selecting a single item from the array and reporting that orientation; if the error is greater than the difference score, then there is some substantial error in participants’ ability to accurately report the orientation of the array.

### Results

**Fig. 6:**
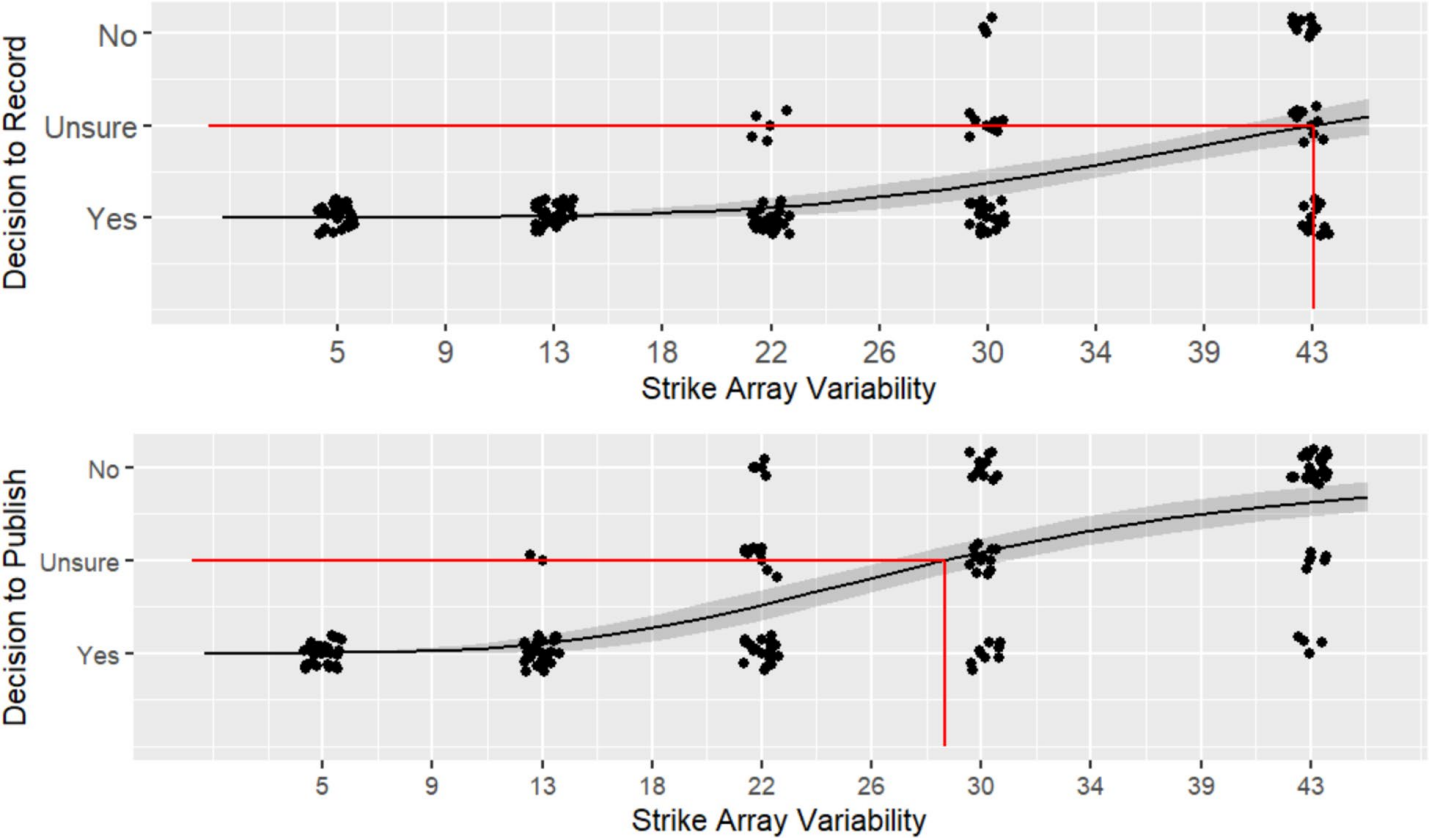
Willingness to record (top) and publish (bottom) across strike array variability level for Experiment 2. Participant response data, represented by the black dots, is jittered for ease of viewing. A sigmoid curve is fit to the response data, represented by the black line, with a 95% confidence interval, represented by the gray shadow around the line. The inflection point of the curve, represented by the red line, indicates the strike array variability level where participants were Unsure, shifting from predominantly Yes responses to predominantly No responses

For **H1,** a lack of fit test was non-significant for both willingness to record (*F* (3) = 0.033, *p* = 0.992), and willingness to publish (*F* (3) = 0.247, *p* = 0.863), indicating the hypothesis is supported and the log-logistic sigmoid curve is a good fit for the data (see Fig. 6). For willingness to record, the RSE was 0.48 (148 degrees of freedom), and for willingness to publish the RSE was 0.57 (147 degrees of freedom).

For **H2**, we compared the inflection point of the curves at which willingness to record and publish began to decrease. Similar to Experiment 1, results supported the hypothesis that the threshold for participants’ willingness to record (*c* = 42.84) would be greater (*t* (49) = 6.63, *p* < 0.001, *d* = 1.71) then their willingness to publish (*c* = 28.79), indicating participants were more tolerant of variability when recording, as opposed to publishing data. Compared to Experiment 1, these inflection point values are notably higher and indicate that participants in Experiment 2 have a greater tolerance for variability, e.g., participants were willing to record data at all levels of variability. The slope at the inflection point for willingness to record (*b* = −4.16) was comparable (*t* (51) = −0.41, *p* = 0.343, *d* = −0.1) to the slope at the inflection point for willingness to publish (*b* = −3.77), and both were much larger than those seen in Experiment 1, indicating participants in Experiment 2 were in better agreement about the level of variability at which data become unrecordable and unpublishable.

Participants’ average subjective rating of variability at the respective thresholds was greater for the decision to record (*M* = 60.23, *SD* = 19.41) than the decision to publish (*M* = 46.87, *SD* = 18.86). As in Experiment 1, this supports that participants are more tolerant of variability when recording data than when publishing. However, the mean subjective variability ratings in Experiment 2 are around 10 points lower than in Experiment 1. This further supports that participants in this experiment are less conservative in their treatment of highly variable data.

**Fig. 7:**
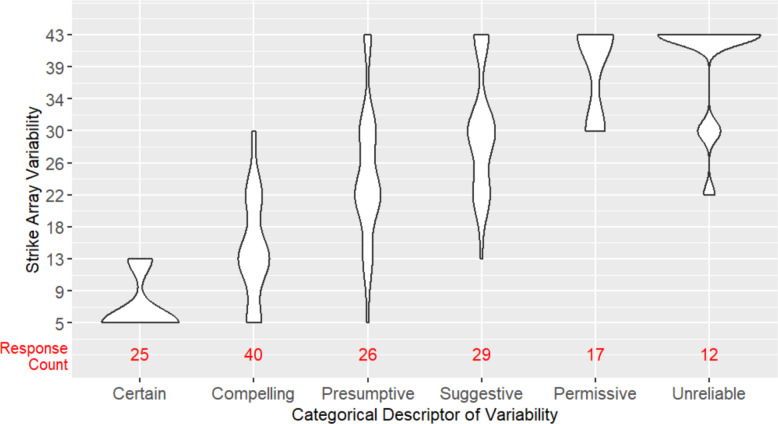
Categorical descriptors of variability used to label strike array variability levels for Experiment 2. The width of each violin plot shows the approximate relative frequency of descriptor selections at that level of strike array variability. Response counts (in red) give the absolute frequency of descriptor selection

For **H3**, we explored the agreement among participants in the strike array variability associated with each categorical descriptor of variability. As in Experiment 1, median strike array variability increases progressively moving from Certain to Unreliable (see Fig. 7). Overall, participants in Experiment 2 have better agreement in the strike array variability associated with each categorical descriptor than they did in Experiment 1. The linear regression analysis showed increasing strike array variability significantly predicted the selection of terms moving from Certain to Unreliable (*β* = −0.10, *p* < 0.001), accounting for 68% of the variation in responses (*F*(1, 147) = 318.9, *p* < 0.001), compared to just 43% of the variance in Experiment 1. This provides support for the hypothesis that there would be good agreement in the strike array variability associated with each categorical descriptor; participants in Experiment 2 are in particularly good agreement about lower levels (Certain, Compelling) and higher levels (Permissive, Unreliable) of uncertainty, but differ in the array variability they associate with moderate uncertainty (Presumptive, Suggestive). On aggregate, participants tend to report that they would publish Certain data and Compelling data (63/65, 96.9% Yes responses to willingness to publish question), and would not publish Permissive and Unreliable data (24/29, 82.8% No responses to willingness to publish question). These publish rates are comparable to those seen in Experiment 1, but the distribution of responses across the different descriptors is much more evenly spread, whereas in Experiment 1 there was disproportionate use of high uncertainty terms.

For **H4**, we evaluated participants’ error in estimated strike orientation across different strike array variability levels and based on their willingness to publish arrays. Participants’ average estimate error was significantly greater (*t* (820) = 3.29, *p* = 0.001, *d* = 0.21) for arrays exceeding the threshold of variability for publication (*M* = 15.08, *SD* = 17.86) versus those arrays below the threshold that the majority of participants indicated they would publish (*M* = 11.44, *SD* = 16.45). A simple linear regression analysis also showed increasing strike array variability significantly predicted average estimate error, accounting for 37% of the variation in responses (*F* (1, 28) = 16.62, *p* < 0.001). For every one degree increase in strike array variability, participants’ predicted mean degrees of error increased by 0.20 (*p* < 0.001). Taken together, these results support the hypothesis that average estimate error would be greater within the strike arrays with higher levels of variability that participants choose not to publish.

**Fig. 8:**
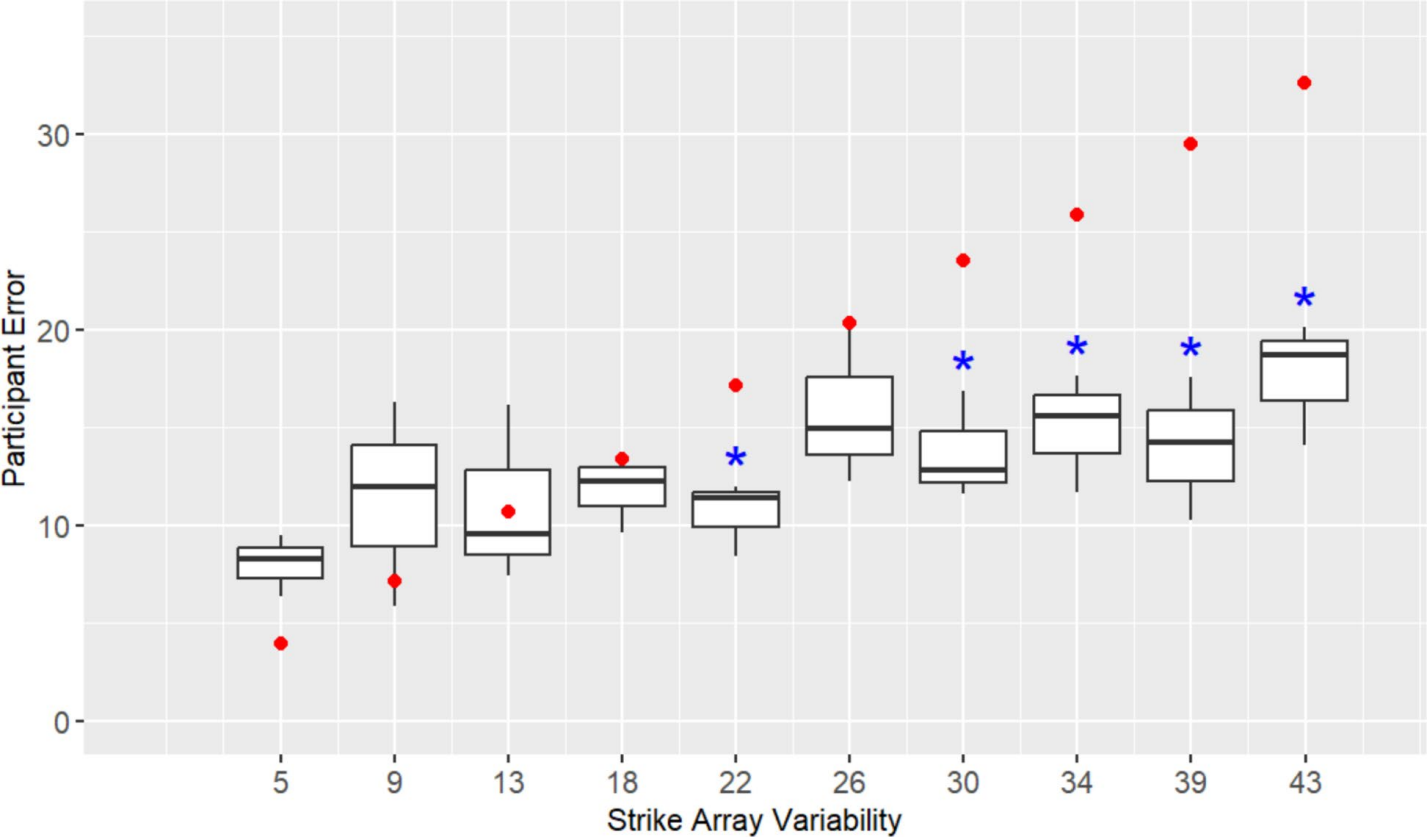
Participants’ orientation estimate error across strike array variability levels in Experiment 2. Dots (in red) at each strike array variability level mark the difference score (between the true average orientation of a strike array and the orientation of each individual strike), with stars (in blue) used to indicate where error significantly differs from the difference score

For **H5**, we compared participants’ average estimate error to the average difference score across different levels of strike array variability (see Fig. 8). The difference score reflects the expected error if one were to estimate the array by choosing an item from it at random and reporting that item’s orientation. As hypothesized, the difference score error was greater than participants’ estimate error, but only at the higher levels of strike array variability: 21.8° (*t* (2) = −6.27, *p* = 0.024, *d* = −3.62), 30.2° (*t* (2) = −4.97, *p* = 0.038, *d* = −2.87), 34.4° (*t* (2) = −5.44, *p* = 0.032,* d* = −3.14), 38.6° (*t* (2) = −9.24, *p* = 0.015,* d* = −5.34), 42.8° (*t* (2) = −10.13, *p* = 0.001,* d* = −5.85). This supports that participants are able to aggregate orientations in arrays, even those at high degrees of variability that exceed their threshold for publication. This is important because it indicates that participants could draw meaningful conclusions from variable data that the majority of participants would choose not to publish.

At the lower levels of strike array variability (9.2°, 13.4°, 17.6°, 26.0°) participant error did not significantly differ from the difference score. This could be driven by participants selecting a single item from the array and reporting that orientation. However, the fact that participants’ are apparently able to aggregate items from arrays with greater degrees of variability suggests an alternative, namely, that participants have some significant fixed error in reporting orientation in our experiment (~ 10°, as shown in Fig. 8). This fixed error could be the result of the measurement tool we provided participants for making the visual estimate of average strike orientation: a compass which had ticks every 5°. This interpretation is supported by participant error being greater than the difference score at 5° of strike array variability (*t* (2) = 4.28, *p* = 0.05, *d* = 2.47).

### Discussion

The results show participants from Experiment 2 generally have a lower-criterion tolerance of variability than those from Experiment 1. Most participants in Experiment 2 were willing to record data even at the highest level of variability (~ 42°) and were willing to publish data up to ~ 28° variability: compare this to participants in Experiment 1 who were only willing to publish data up to ~ 10° variability. Participants in Experiment 2 also had lower subjective ratings of variability at the record/publish thresholds than participants in Experiment 1. Across the two experiments, we believe we capture a broadly representative sample of the geology community, with scientists who differ in tolerance of variability over a spectrum of low-criterion (i.e., willing to record high variability data and often publish) to high-criterion (i.e., never willing to publish high variability data and often not willing to record).

Regardless of where scientists fall on this spectrum, our results suggest that most scientists are able to make roughly accurate estimations of variable data. While participants' error in estimates increased with increasing variability, their error at higher degrees of variability was less than the difference score between the true average orientation of a strike array and the orientation of each individual strike. This would suggest that participants are not just reporting the orientation of a single item in the array but are engaging in some kind of visual averaging that results in estimates with error ranging between ~ 10° to 20°. Taken together, these results imply there may be scientists who avoid recording or publishing variable data, despite being able to draw meaningful conclusions from such data.

In Experiment 2, we may have unintentionally intervened to shift participants to a lower-criterion tolerance of variability by having them make strike orientation estimates from variable strike-dip arrays *prior* to reporting their willingness to publish/record those arrays. It is possible that the act of estimation made salient to scientists that they were, in fact, decently good at estimating trends from highly variable data, which subsequently made them more likely to report that they would be willing to record/publish it. Thus, the tendency for participants to have a high-criterion tolerance of variability in Experiment 1 and a lower-criterion in Experiment 2 could be driven more by the study procedure and less by individual differences across samples. If this is the case, we would expect that changing the procedure order—so participants make publish/record judgments before estimating orientation—would result in participants having a higher-criterion tolerance, akin to Experiment 1. We test this in Experiment 3.

## Experiment 3

The goal of Experiment 3 was to assess whether the act of making orientation estimates before reporting willingness to record/publish data drove participants in Experiment 2 to appear to have a lower-criterion tolerance of variable data than participants in Experiment 1 (who did not make estimates).

### Methods

#### Design and variables

This experiment used the same design and variables as Experiment 2, but changed the procedure order so that participants made judgments of willingness to record/publish prior to making orientation estimates.

#### Hypotheses

We had one additional hypothesis to those stated in Experiment 2 (**H1**—**H5**), relevant to the change in procedure order in Experiment 3. We hypothesized that participants would be generally conservative in their treatment of high variability data, choosing not to record or publish data at thresholds of variability lower than Experiment 2 and more in line with the thresholds seen in Experiment 1 (**H6**).

#### Materials and procedure

As in the previous experiments, participation occurred through a web-based Qualtrics survey and participants were first asked to provide informed consent and confirm that they were completing the survey on a desktop or laptop computer (and not a mobile device). The survey was nearly identical to the one used in Experiment 2, but the procedure order was changed so judgments of willingness to record and publish occurred prior to orientation estimates, and participants were asked to make fewer orientation estimates in order to reduce the amount of time required to complete the study and increase the chances experts would be willing to participate. A subset of the same arrays of strike-dip symbols were used as Experiment 2: 5 variability values that encompassed the range used in Experiment 1 and 2 (5°, 13.4°, 21.8°, 30.2°, and 42.8°), each in 3 starting orientations, for a total of 15 arrays.. Participants completed the same demographic questions as previous experiments.

#### Participants

Twenty-two experts in a variety of geology fields participated in this experiment and were included in the analysis. All participants had at least a bachelor’s degree and 80% had at least 5 years of experience post-bachelor’s (*M* = 20.4, *SD* = 14.45). The majority of participants (*n* = 12) reported specializing in structural geology and tectonics, with a few (*n* = 5) specializing in mineralogy, geochemistry, petrology, and volcanology, and no more than 2 specializing in any other subarea. Most participants also reported primarily working in academia (*n* = 13), 5 reported primarily working for a government entity, 1 reported working in industry, and 1 reported working in both industry and academia. Participants were recruited using the same methods as previous experiments. As participation was anonymous, we have no way of determining whether any of the participants from Experiments 1 and 2 also participated in Experiment 3. Data were collected from November 2024 to January 2025.

#### Data analysis

We used the same data cleaning procedure as Experiment 2, removing participants based on the following criteria: they were not 18 years of age or older (none excluded), they did not complete the questions on a laptop or computer (8 participants excluded), they did not complete at least 75% of the survey questions (6 participants excluded), they had a mean negative slope for their subjective rating of variability across strike array variability levels (3 participants excluded), more than 1/2 of their estimations of overall strike array orientation had an error that differed ± 2 standard deviations from the mean of actual strike array orientations (1 participant excluded). We removed 18 participants through this cleaning procedure from the 40 total who participated, and ran all subsequent analyses on the 22 remaining participants. All other aspects of analysis were identical to those described in Experiment 2.

### Results

**Fig. 9:**
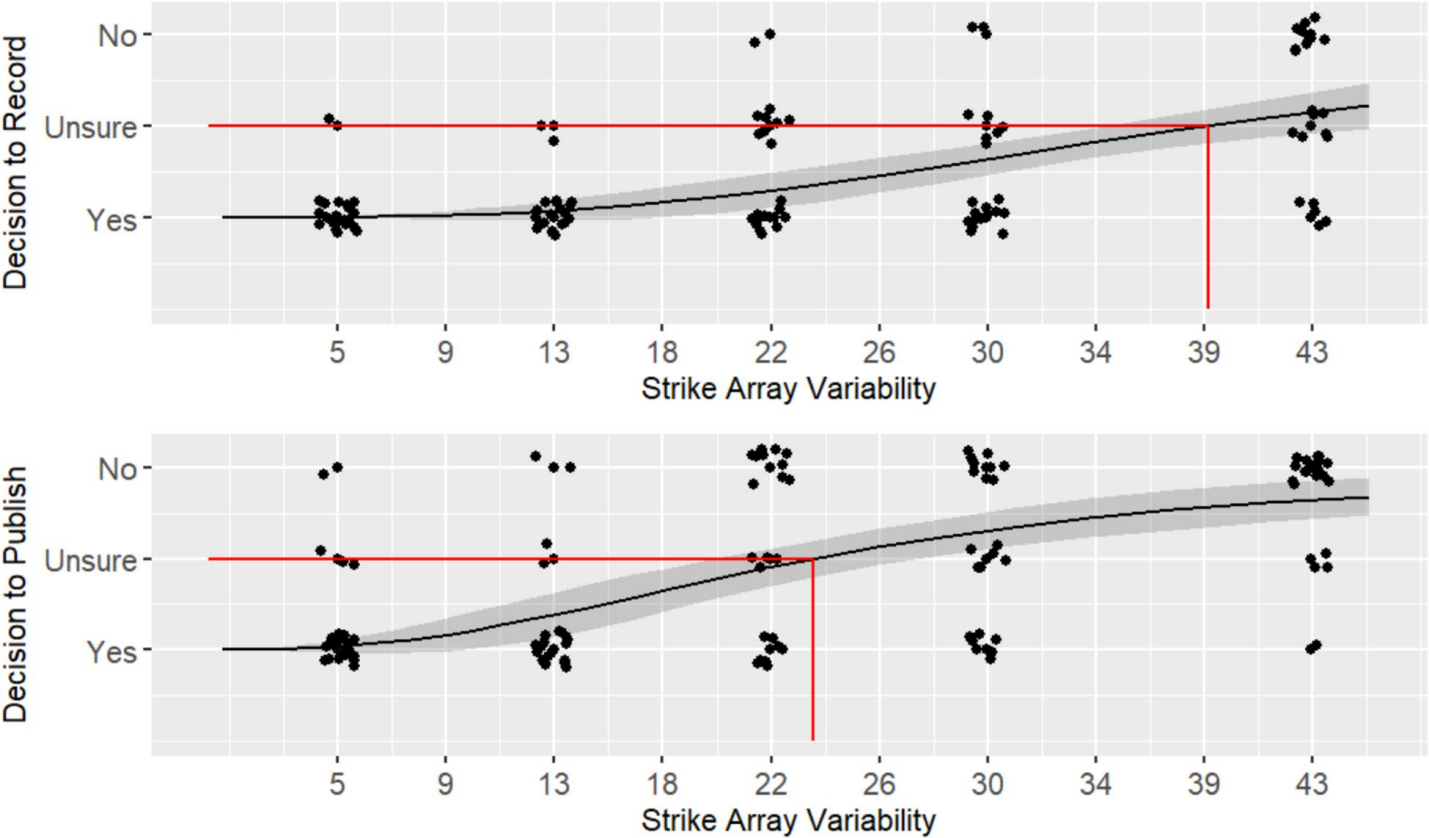
Willingness to record (top) and publish (bottom) across strike array variability level for Experiment 3. Participant response data, represented by the black dots, is jittered for ease of viewing. A sigmoid curve is fit to the response data, represented by the black line, with a 95% confidence interval, represented by the gray shadow around the line. The inflection point of the curve, represented by the red line, indicates the strike array variability level where participants were Unsure, shifting from predominantly Yes responses to predominantly No responses

For **H1,** a lack of fit test was non-significant for both willingness to record (*F* (3) = 1.53, *p* = 0.211), and willingness to publish (*F* (3) = 2.12, *p* = 0.10), indicating the hypothesis is supported and the log-logistic sigmoid curve is a good fit for the data (see Fig. 9). For willingness to record, the RSE was 0.57 (108 degrees of freedom), and for willingness to publish the RSE was 0.73 (108 degrees of freedom).

For **H2**, we compared the inflection point of the curves at which willingness to record and publish began to decrease. As in the previous experiments, results supported the hypothesis that the threshold for participants’ willingness to record (*c* = 38.80) would be greater (*t* (39) = 5.04, *p* < 0.001, *d* = 1.52) then their willingness to publish (*c* = 23.44,), indicating participants were more tolerant of variability when recording, as opposed to publishing data. Relevant to **H6**, the inflection point values reflect a tolerance for high variability data, more in-line with the participants from Experiment 2 than those from Experiment 1. Thus, having participants make publish/record judgments *before* estimating strike array variability (rather than after, as in Experiment 2) did not result in participants being more conservative in treatment of high variability data. The slope at the inflection point for willingness to record (*b* = −3.01) was comparable (*t* (39) = −0.5, *p* = 0.311, *d* = −0.15) to the slope at the inflection point for willingness to publish (*b* = −2.54). Agreement about the level of variability at which data became unrecordable and unpublishable was not as strong as in Experiment 2, but generally better than Experiment 1.

Participants’ average subjective rating of variability at the respective thresholds was greater for the decision to record (*M* = 72.41, *SD* = 18.06) than the decision to publish (*M* = 47.36, *SD* = 23.79). As in the previous experiments, this supports that participants are less conservative in their treatment of highly variable data when recording versus publishing. The mean subjective variability ratings are comparable to Experiment 2 (lower than Experiment 1), further supporting that participants in this experiment are tolerant of high variability data.

**Fig. 10:**
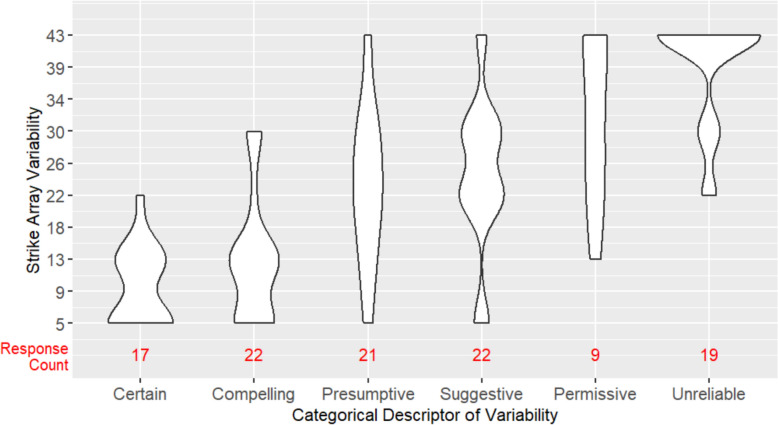
Strike array variability levels associated with categorical descriptors of variability for Experiment 3. The width of each violin plot shows the approximate frequency of descriptor selections at that level of strike array variability

For **H3**, we explored the agreement among participants in the strike array variability associated with each categorical descriptor of variability. As in the previous experiments, median strike array variability increases progressively moving from Certain to Unreliable (see Fig. 10), but overall, participants in Experiment 3 have slightly lower agreement in the strike array variability associated with each categorical descriptor than they did in Experiment 2. The linear regression analysis showed increasing strike array variability significantly predicted the selection of terms moving from Certain to Unreliable (*β* = −0.09, *p* < 0.001), accounting for 55% of the variation in responses (*F*(1, 108) = 332.4, *p* < 0.001), compared to 68% of the variance in Experiment 2, and 43% in Experiment 1. This provides support for the hypothesis that there would be good agreement in the strike array variability associated with each categorical descriptor; participants in Experiment 3 are in particularly good agreement about lower levels (Certain, Compelling) and the highest level (Unreliable) of uncertainty, but differ in the array variability they associate with moderate uncertainty (Presumptive, Suggestive, Permissive). On aggregate, participants tend to report that they would publish Certain data and Compelling data (36/39, 92.3% Yes responses to willingness to publish question), and would not publish Permissive and Unreliable data (24/28, 85.7% No responses to willingness to publish question). These publish rates are comparable to those seen in Experiment 1 and 2, with a fairly even distribution of responses across descriptors.

For **H4**, we evaluated participants’ error in estimated strike orientation across different strike array variability levels and based on their willingness to publish arrays. As in Experiment 2, results supported the hypothesis. Participants’ average estimate error was significantly greater (*t* (225) = 3.29, *p* < 0.001, *d* = 0.41) for arrays exceeding the threshold of variability for publication (*M* = 18.17, *SD* = 21.98) versus those arrays below the threshold (*M* = 10.33, *SD* = 16.75), and increasing strike array variability significantly predicted average estimate error, accounting for 69% of the variation in responses (*F* (1, 13) = 29.4, *p* < 0.001). For every one degree increase in strike array variability, participants’ predicted mean degrees of error increased by 0.30 (*p* < 0.001), roughly the same rate as in Experiment 2.

**Fig. 11:**
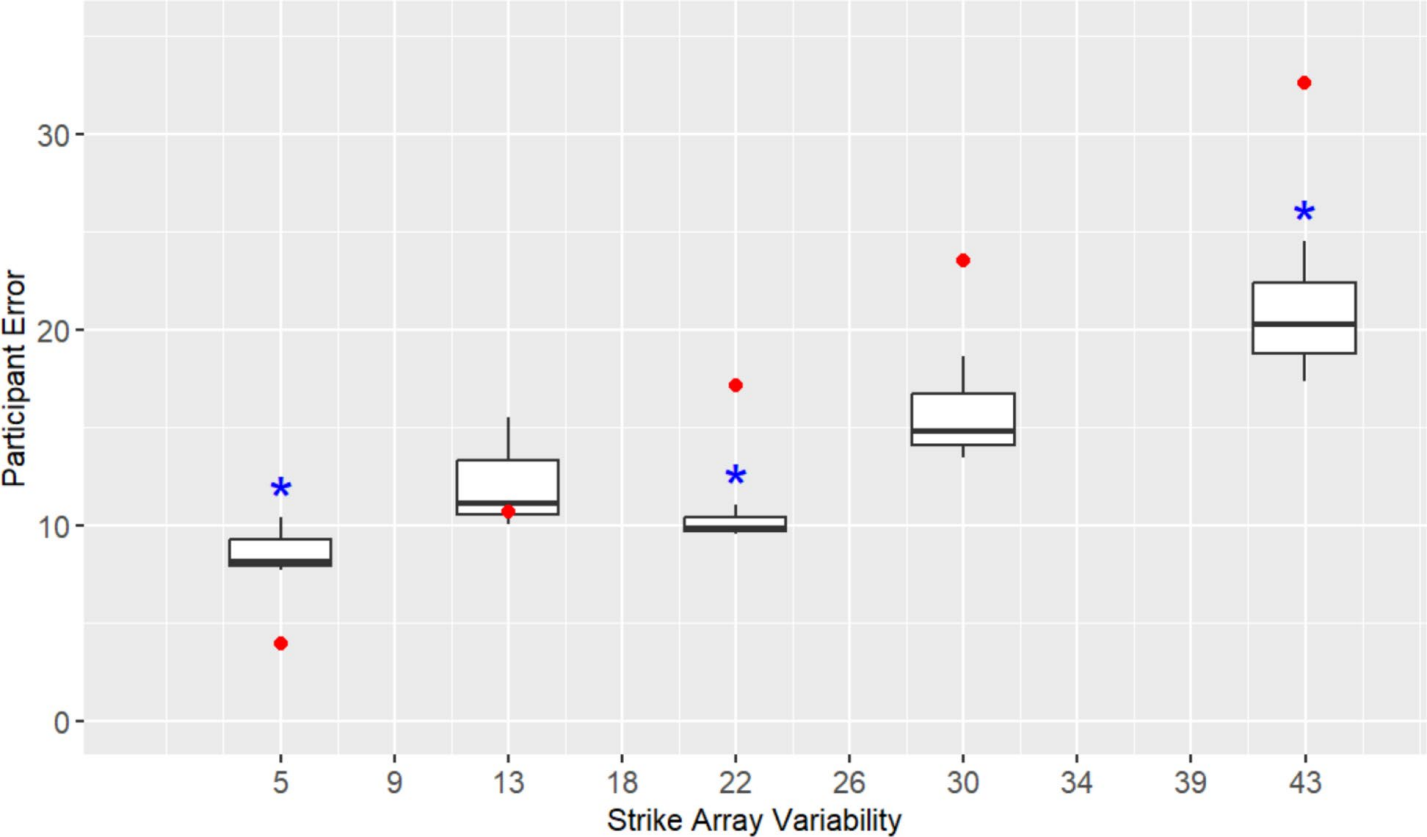
Participants’ orientation estimate error across strike array variability levels in Experiment 3. Dots (in red) at each strike array variability level mark the difference score (between the true average orientation of a strike array and the orientation of each individual strike), with stars (in blue) used to indicate where error significantly differs from the difference score

For **H5**, we compared participants’ average estimate error to the average difference score across different levels of strike array variability (see Fig. 11). Similar to Experiment 2, participant error was lower than the difference score at most of the higher levels of strike array variability—21.8° (*t* (2) = −15.07, *p* = 0.004, *d* = −8.70), 30.2° (*t* (2) = −4.06, *p* = 0.056,* d* = −2.34), 42.8° (*t* (2) = −6.55, *p* = 0.021,* d* = −3.78)—suggesting participants are able to extrapolate orientations from arrays with variability beyond levels that they would typically publish or record. Error did not differ from the difference score at 13.4°, and was greater than the difference score at 5° (*t* (2) = 5.51, *p* = 0.031, *d* = 3.18). These results are broadly consistent with Experiment 2, suggesting participants have a fixed error in reporting orientation that we believe to be the result of the compass we provided them for making visual estimates.

### Discussion

The results from Experiment 3 confirm that differences in the criterion tolerance of variable data between participants from Experiment 1 and Experiment 2 were not driven by the study procedure itself. Rather, there are apparent individual differences in criterion, and we happened to recruit a sample of geologists in Experiment 1 with a higher-criterion, whereas geologists in Experiments 2 and 3 tended to have a lower-criterion. In the general discussion, we explore demographic differences between participant samples that could help to explain individual differences in criterion tolerance of variability.

Experiment 3 results provide additional evidence that there may be scientists who avoid recording or publishing variable data, despite being able to draw meaningful conclusions from such data. As in Experiment 2, participants in Experiment 3 were able to make decently good visual estimates of orientations from arrays with variability beyond levels that most reported being willing to publish or record. The implications of this finding are discussed in detail in the next section.

#### General discussion

Across three experiments, we demonstrate geologists (i) have a tendency to respond to variable data in strike-dip data arrays by choosing not to record or publish it, but (ii) are capable of managing variability by making visual estimates of the representative strike orientation. In the context of a data gathering workflow, these are analogous to ignoring and minimizing variability and are consistent with the most common strategies adopted by professional data workers, as described in Boukhelifa et al. (2017). When scientists are able to minimize (aggregate) variability, but instead choose to ignore data that are highly variable (not publish/record), this is an active data variability management strategy. Management strategies reflect an individual scientist’s criterion tolerance of variability. Those geologists who have a higher-criterion are less likely to retain noisy data than those who have a lower-criterion; we saw large individual differences in criterion, while participants across experiments were all generally good at making accurate estimates of orientation.

Participant differences in criterion tolerance of variability could be driven by disciplinary specialization, or by some other individual preference. Given that the majority, 68%, of participants in our experiments reported a specialization of structural geology and tectonics, our results point to a stronger role for individual preference. In fact, because structural geology is a primarily observational subdiscipline, we might have expected participants to trend toward a lower-criterion tolerance due to their experience working in intrinsically high variability environments. That we saw more spread in tolerance of variability among structural geologists presents interesting considerations for how we think about data variability management within geology, since there is likely a range in variability across subdisciplines. We conjecture that field-oriented, observational subdisciplines experience high variability and more experimentally oriented subdisciplines, such as petrology or mineralogy, experience less variability. Are scientists used to working with less variable data more conservative in their tolerance of variability? Our current sample was too small to attempt to characterize differences across subdisciplines in variable data management decisions. However, in considering the scope of applicability of these findings it is worth noting that other observational sciences (e.g., astronomy, archeology, ecology) may face variable data and analogous questions about recording and sharing. Within psychology there are subdisciplines that often work with observational data (e.g., developmental and clinical). Although there is awareness of potential for bias and best sampling practices have been recommended (Bornstein et al., 2013), it would be worth considering how researchers in these areas manage decisions about variable data.

The finding that geologists in our sample could effectively judge trends from highly variable data is consistent with the common field practice of selecting a representative rock outcrop to record as data. Although research shows laypeople are generally good at estimating collective trends from an ensemble (Parkes et al., 2001), we believe this perceptual skill is particularly well-developed through training and experience in geology. We ran a simplified pilot version of this task with undergraduate psychology students who were predominantly at chance in judging the overall trend in arrays. Considering the quality of data in geology, these findings suggest the extant geology data are representative of the world and thus a solid foundation for practice. These findings also point to an opportunity for the field: to share all recorded data, even if the variability exceeds one’s threshold for publication, understanding that in combination with others’ variable data (and given enough data), meaningful scientific conclusions might be drawn. A reluctance to record variable data may reflect logistical time constraints in the collection of field data: when faced with limited time and resources, efforts should be first devoted to areas where meaningful, systematic trends can be confidently observed.

It is important to note that, prior to the introduction of digital data, geologists had no way of conveying data quality to other practitioners with geological maps. Thus, geologists were faced with a “threshold” problem, because the data were either included or not. With the advent of digital databases and field apps to quickly record observations (Walker et al., 2019), the opportunity to share all recorded data may be realized. The lower a geologist’s threshold for recording variable data the better such data will reflect variability in the world. Such datasets would be valuable for both confirmatory statistical analyses, but also allow new types of exploratory analyses to be conducted that can reveal higher-order regularities at the level of variability (analogous to individual differences approaches to understanding complex systems in developmental psychology). Thus, these findings may serve as a stimulus to the geology science community to consider opportunities for changes in disciplinary training and practice with the increasing availability of digital tools in the field. An analogous effort has already been made in empirical psychological science research to encourage adoption of public study registries and data repositories (Soderberg, 2018) to address the file-drawer problem of not submitting null results for publication (Moniz et al., 2025).

The authors are part of an effort to promote geologists recording their uncertainty when they collect data (Nelson et al., 2023; Tikoff et al., 2023). We have argued that this is valuable meta-data to share with colleagues that previously existed only in field notebooks. These data reflect the mental state of the geologist and could assist in evaluating how well data support a geological model of the field area. Sharing these data supports trust in data, robust model evaluation, transparency in model development, and increases the usability of data by people who have not traveled to the field area to assess the nature of variability in the rocks in that location. To support uncertainty communication, we advocated for a workflow that included recording uncertainty using a six-item scale of qualitative categories: Unreliable, Permissive, Suggestive, Presumptive, Compelling, and Certain. While multiple factors could influence the uncertainty, one important one would be the perceived variability in an area. In the current study, we used the scale to collect qualitative judgments of variability as an initial assessment of the reliability of a scale with these six categories. There was reasonably good reliability in the correlations between objective variance of the array and qualitative category assignment (*r*^*2*^ ranged from 0.43 to 0.68) across the three experiments. To improve agreement and reduce individual differences in scale usage, we propose (i) developing idealized variability examples as employed for estimating disciplinary measures such as grain size or percent composition and (ii) community discussions to establish disciplinary standards for spatial data (e.g., strike dip and position) and non-spatial data (e.g., lithology).

## Limitations

A limitation of the current work is that we cannot specify the impact that changes in study stimuli between experiments had on participants tolerance of variability and judgment behavior. It was necessary to generate a new, larger set of strike-dip arrays for Experiments 2 and 3 that was in most parameters identical to the one used in Experiment 1 with different levels of strike variability, but also included different starting orientations to test orientation estimation, requiring an increase in set size (from 10 arrays to 30). We opted to have participants make judgments of willingness to publish and record on only a subset of 5 arrays in Experiments 2/3, with variability values that encompassed the range used in Experiment 1, to keep the duration of the experiment reasonable for successful recruitment of the expert geologist population of study. For the same reason, in Experiment 3 we reduced the array set size from 30 to 15. We believe the effect of reducing the number of array judgments was nonexistent or negligible, where an effect to have occurred it would be expected to make participants less tolerant of variability (because with only a subset of arrays they see bigger jumps in the level of variability), but the opposite pattern is observed (more tolerant of variability with subset of arrays). We cannot rule out the effect that changing set size had on performance. The sample of experts in Experiment 2 that saw the most arrays tended to be the most tolerant of variability, while the sample in Experiment 1 that saw the fewest arrays tended to be the least tolerant of variability. However, we still saw individual differences in tolerance of variability within each experiment, suggesting any effect of array set size on tolerance of variability is secondary to the preexisting level of tolerance participants bring with them to the experiment.

Another limitation is that our recruitment procedures led to sampling bias, with overrepresentation of the sub-disciplinary specialization of structural geology and tectonics and positions in academia. To better understand individual differences in tolerance of variability, it is essential to include scientists from a wider array of professional experience, and to test whether results are generalizable to the broader disciplinary geology community, or other observation-based science communities (as discussed in the preceding Section). We faced increasing attrition rates across the three study recruitment windows, which can be partially attributed to tapping the same network of geologists through our recruitment methods, but is also likely due to the stimulus set increasing and experiments taking longer. However, we were still sufficiently powered to detect the large magnitude effects of variability on judgments to record and publish and accuracy of orientation estimates.

## Conclusions

Geologists excel at making observations and extracting trends obscured by variability. However, individual differences in their criterion tolerance of variability impact willingness to record observations, turning them into data which may be communicated to the science community through publication. Choosing not to record variable data is a good strategy in conditions where time and resources are constrained and it is better to focus on systematic trends that can be confidently observed—but it presents a missed opportunity, since given enough variable data (e.g., through a digital public repository), meaningful scientific conclusions can be drawn. This study acts as a stimulus to the geology science community to consider changes in disciplinary training and practice surrounding the recording of variable field data and highlights the value of digital tools for recording, storing, and communicating data.

## Data availability and materials

The materials, data, and data analysis scripts used during the current study are available in the OSF repository, 10.17605/OSF.IO/S2JD6.

## Abbreviation

RSE: Residual standard error
